# Insights into the mechanism of idiopathic left ventricular tachycardia: a case report and literature review

**DOI:** 10.1186/s40001-015-0156-y

**Published:** 2015-09-17

**Authors:** Paul Puie, Gabriel Cismaru, Lucian Muresan, Radu Rosu, Mihai Puiu, Marius Andronache, Gabriel Gusetu, Roxana Matuz, Petru-Adrian Mircea, Dana Pop, Dumitru Zdrenghea

**Affiliations:** Department of Cardiology, Rehabilitation Hospital, “Iuliu Hatieganu” University of Medicine and Pharmacy, 46-50 Viilor Street, 400347 Cluj-Napoca, Romania; Department of Electrophysiology, Institut Lorrain du Coeur et des Vaisseaux «Louis Mathieu», CHU de Nancy, Cluj-Napoca, France; Department of Internal Medicine, Medical Clinic No 1, “Iuliu Hatieganu”, University of Medicine and Pharmacy, Cluj-Napoca, Romania

**Keywords:** Ventricular tachycardia, Left posterior fascicle, Ablation, Mapping, Amiodarone

## Abstract

Left ventricular posterior fascicular tachycardia (LVPFT) is an idiopathic form of VT characterized by right bundle branch block morphology and left axis deviation. The mechanism of LPFVT is thought to be localized reentry close to the posterior fascicle. We present the case of a 24-year-old medical student who was admitted to the emergency department complaining of palpitations. The ECG showed an aspect suggestive of LVPFT. Vagal maneuvers, adenosine and i.v. Metoprolol were ineffective in terminating the arrhythmia. Conversion to sinus rhythm was obtained 10 h later, with i.v Amiodarone. The ECG in sinus rhythm showed left posterior fascicular block. Because antiarrhythmic drugs were not desired by the patient, VT ablation was proposed. The electrophysiological study identified the mechanism of arrhythmia to be reentry using the slowly conducting verapamil-sensitive fibers as the antegrade limb and the posterior fascicle as the retrograde limb. Radiofrequency applications near the posterior fascicle, in the lower half of the interventricular septum, at the junction of the two proximal thirds with the distal third interrupted the tachycardia and made it non-inducible at programmed stimulation. The case is unusual as the patient had a left posterior fascicular block during sinus rhythm before ablation. This demonstrates that the reentry circuit of VT does not need antegrade conduction through the posterior fascicle for perpetuation.

## Background

Fascicular ventricular tachycardia (VT) is an idiopathic form of VT characterized by right bundle branch block morphology and left axis deviation. It occurs predominantly in young males (15–40 years old) [1]. Left posterior fascicular VT (LPFVT) is the most frequent form, encountered in approximately 90% of cases, the other 10% being represented by left anterior fascicular VT and upper septal fascicular VT [2]. The mechanism of LPFVT is believed to be reentry using the slowly conducting verapamil-sensitive fibers as the antegrade limb and the posterior fascicle as the retrograde limb [3]. This type of VT responds well to i.v. verapamil, but the therapy of choice is catheter ablation, due to the variable efficacy of chronic oral verapamil [4].

## Case presentation

**Fig. 1:**
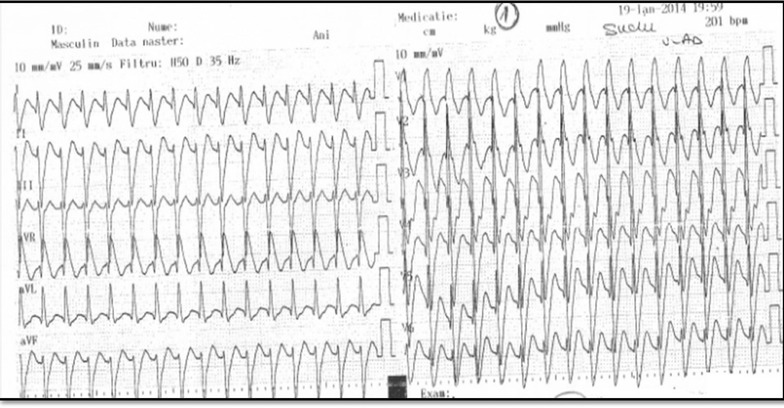
ECG during ventricular tachycardia. ECG shows a monomorphic right bundle branch block tachycardia with a QRS duration of 120 ms (narrower than other forms of VT) right axis deviation, an appearance illustrative for fascicular ventricular tachycardia.

**Fig. 2:**
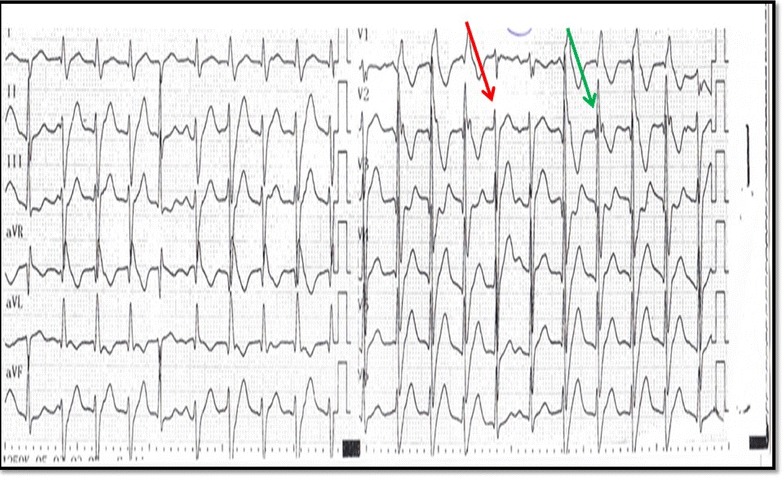
ECG after amiodarone infusion. After slowing the heart rate with amiodarone, ECG shows captures (*red arrow*) and fusion beats (*green arrow*) suggestive of ventricular tachycardia.

**Fig. 3:**
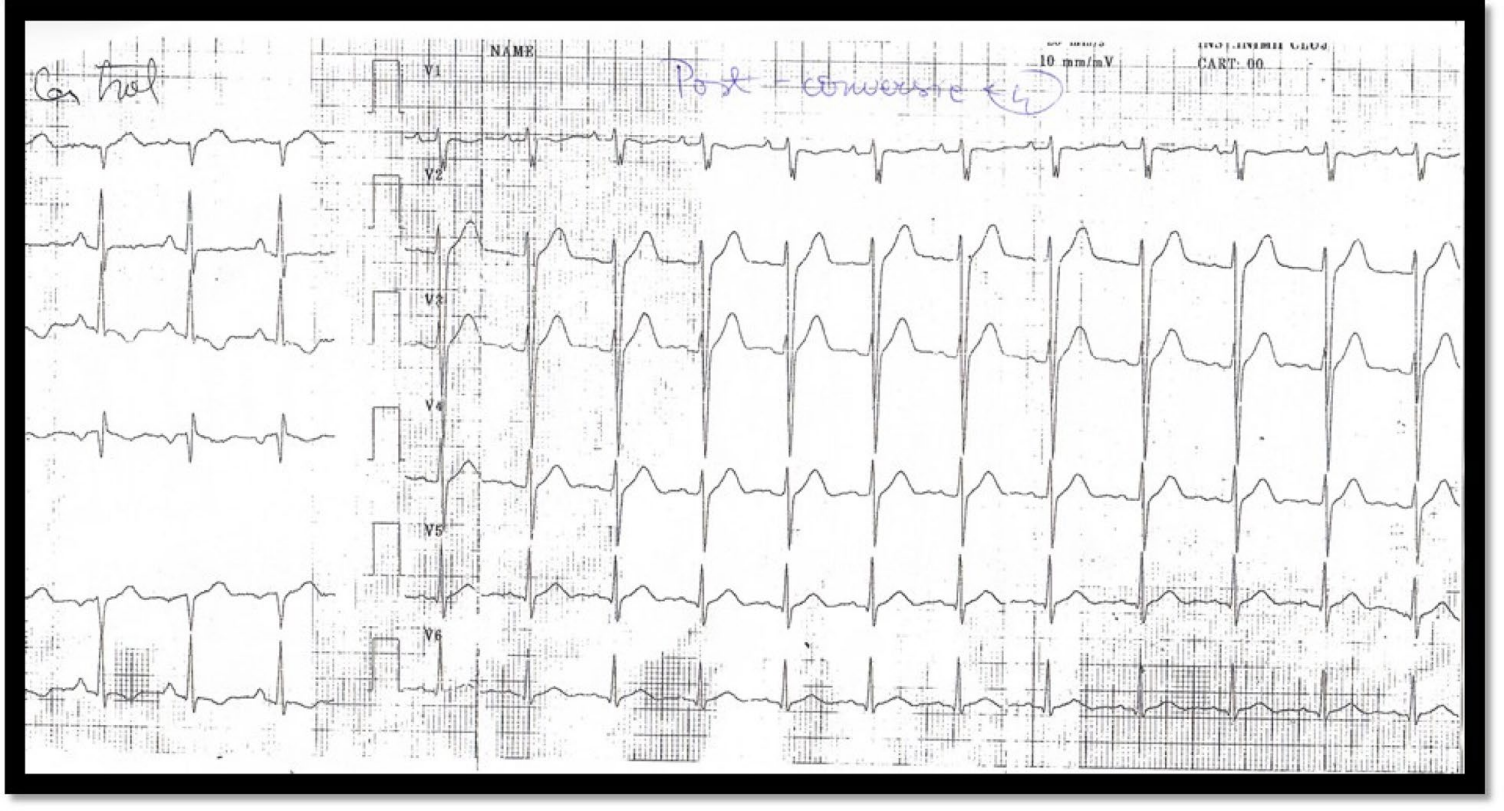
ECG after 10 h of amiodarone infusion. ECG shows conversion to sinus rhythm. In lead I the QRS complex is negative, suggestive of an intrinsic disease of the postero-inferior fascicle of the left branch.

A 24-year-old medical student was admitted to the emergency department due to palpitations triggered by emotional stress (after being on call for 24 h) without hemodynamic instability. The palpitations had begun 1 h prior to his arrival at the hospital. His blood pressure was 130/70 mmHg and no signs of heart failure were present. The 12-lead ECG showed a right bundle branch block (RBBB) wide QRS tachycardia with right axis deviation, an aspect suggesting fascicular VT (Fig. 1). Intravenously adenosine and betablockers were ineffective in terminating the arrhythmia. After intravenous Amiodarone, ventricular captures and fusions beats confirmed the diagnosis of ventricular tachycardia (Fig. 2), which subsequently converted to sinus rhythm 10 h later (Fig. 3). Laboratory values including hemogram, liver function tests, renal function tests, serum electrolytes and TSH were normal. Echocardiography showed a non-dilated left ventricle with normal systolic and diastolic function. A minor anterior mitral valve leaflet prolapse was seen. He was discharged on oral Verapamil 80 mg bid, but the patient refused chronic antiarrhythmic treatment. He was offered a catheter ablation procedure as an alternative, which he accepted.

**Fig. 4:**
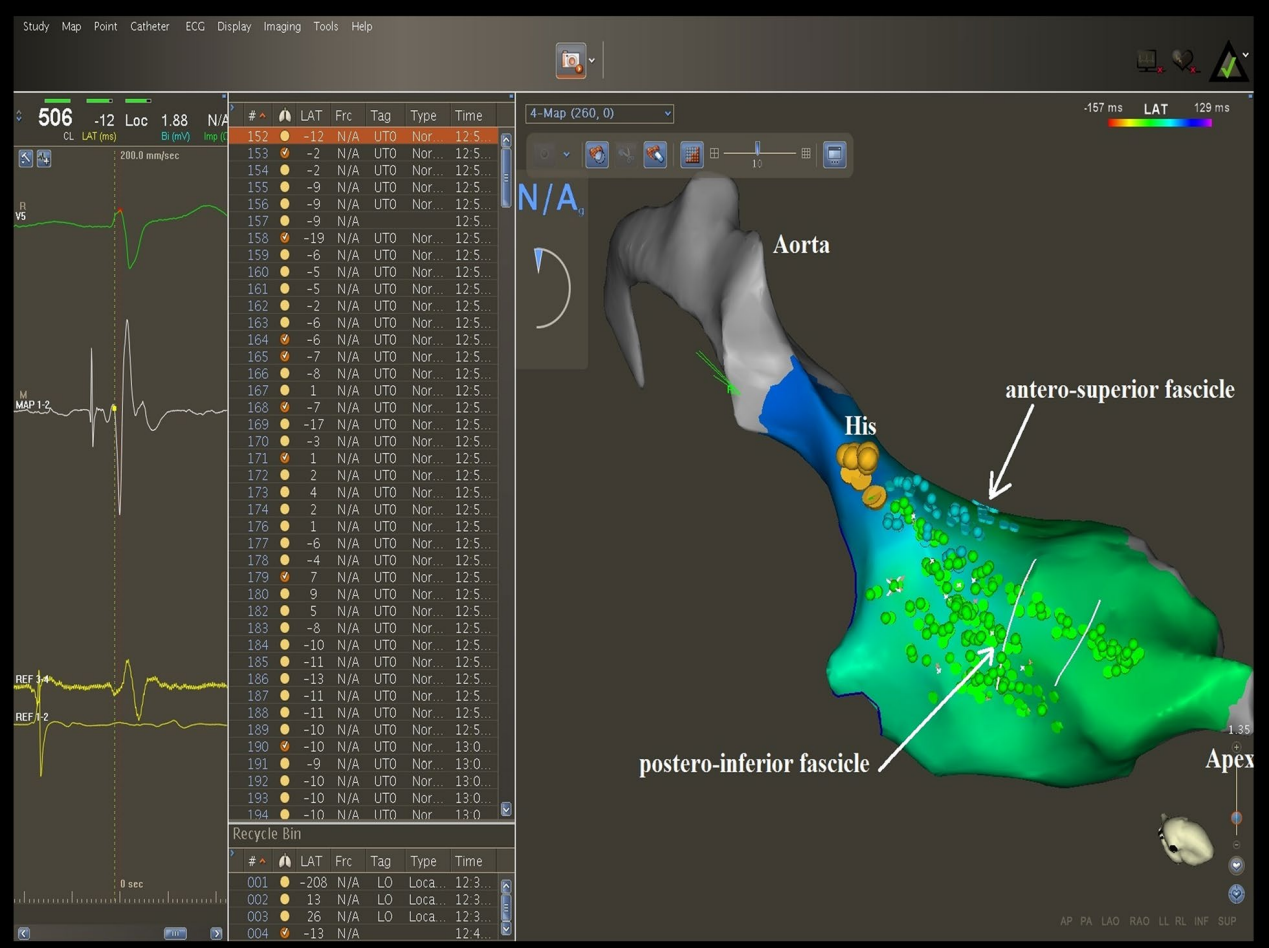
Three dimensional electroanatomical mapping of the left branch of the conduction system. Mapping was done using the Carto 3 system (Biosense Webster). Left ventricular anatomy reconstructed during anatomical mapping; with *gray* the ascending aorta. *Yellow dots* His bundle location (the electrical signal at this level shows a sharp ample potential). *Blue dots* the antero-superior fascicle (the electrical signal at this level shows a sharp small potential). *Green dots* the postero-inferior fascicle (electrical signal at this level shows a sharp small potential). *White lines* the region of the postero-inferior fascicle where the ablation points were targeted.

**Fig. 5:**
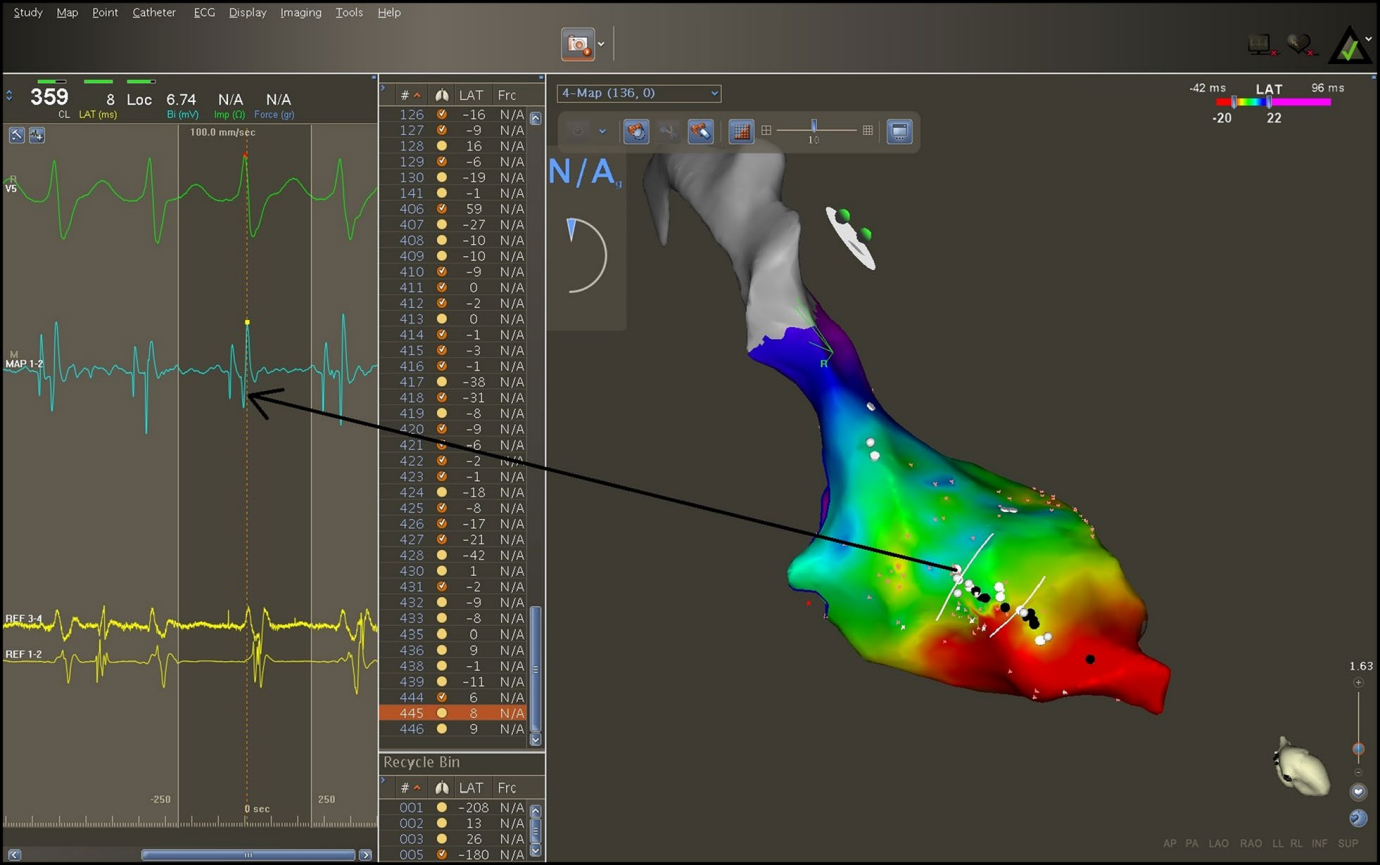
Activation mapping of the left ventricle using the Carto 3 system (Biosense Webster). The *red area* indicates the area of earliest activation. The last region of the left ventricle activated during VT is the latero-basal wall and is indicated by the *blue* and *purple color*. Fast, ample presystolic potentials were recorded at the level of postero-inferior fascicle, which express activation of the local Purkinje network (*white* and *black dots*). Bipolar electrograms obtained at the level of postero-inferior fascicle are indicated by *blue color.*

After obtaining informed consent, an electrophysiological study was performed in a post-absorptive state, using a tridimensional electroanatomical mapping system (CARTO^®^ 3, Biosense Webster). An anatomical map of the left ventricle was initially created, carefully delineating the mitral annulus, the aortic annulus, the conduction system with the His bundle, the left posterior fascicle and the left anterior fascicle (Fig. 4). The electroanatomical bipolar voltage map did not show any area of ventricular scar. Programmed ventricular stimulation was then performed, which induced a wide QRS complex tachycardia with RBBB aspect and right superior axis, with the same cycle length and aspect on the 12 lead ECG compared to his clinical tachycardia. Because the tachycardia was well-tolerated, an activation map of the left ventricle was created. The mechanism of the tachycardia was found to be reentry involving the posterior fascicle, with the exit point situated in the lower half of the interventricular septum, close to the apex. The activation spread from this point to the entire left ventricle. The last region of the left ventricle which was activated was the postero-basal region (Fig. 5).

**Fig. 6:**
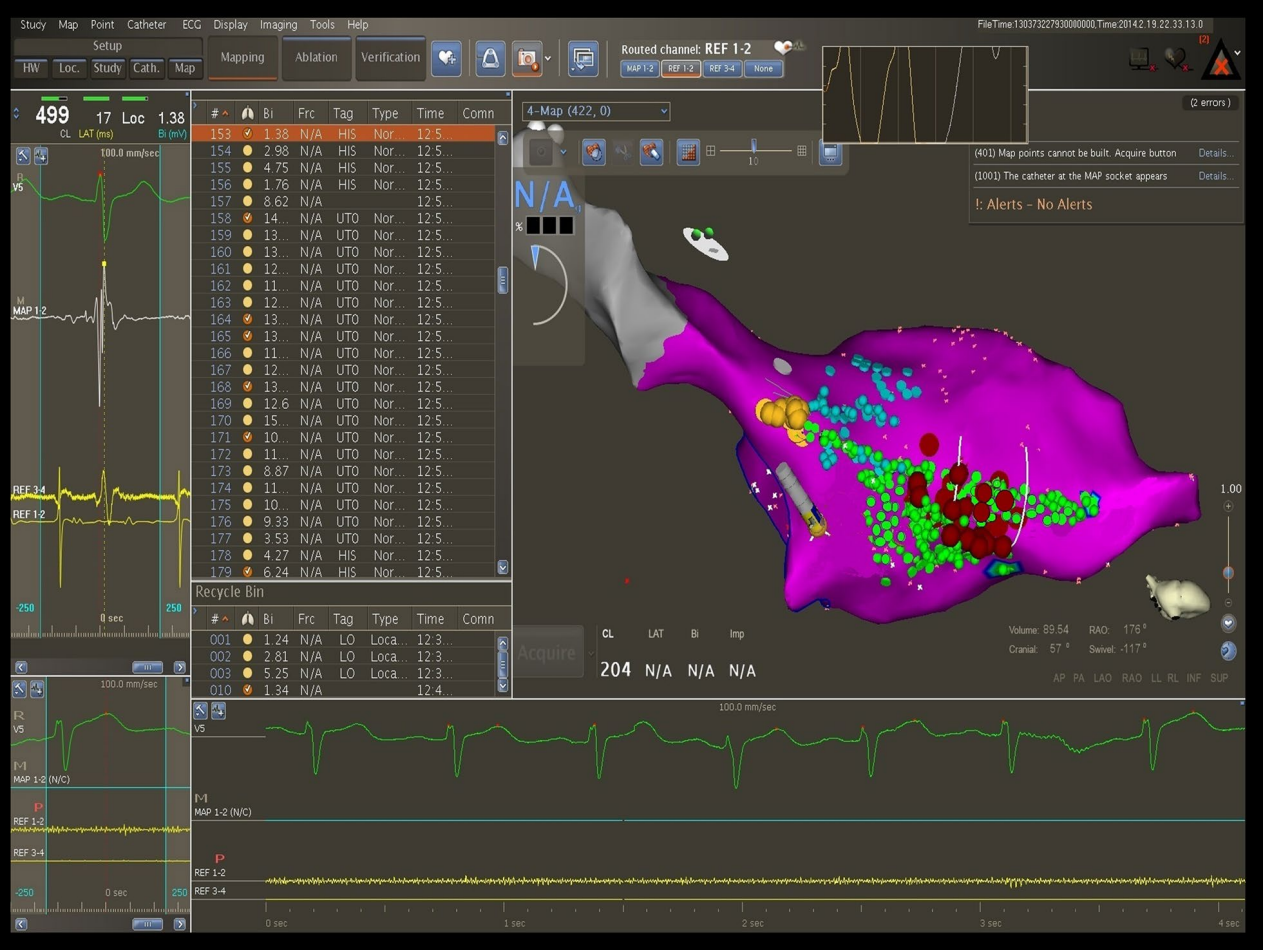
Ablation of the left postero-inferior fascicle using the Carto 3 system (Biosense Webster). The *red dots* indicate ablation points at the level of the postero-inferior fascicle, at the junction of the two proximal thirds with the distal third.

Using a retrograde aortic approach, an irrigated 3.5 mm tip catheter (Navistar Thermocool) was introduced in the left ventricle. Radiofrequency (RF) was applied near the posterior fascicle, in the lower half of the interventricular septum, at the junction of the two proximal thirds with the distal third (Fig. 6), with a power of 30 W and a target temperature of 45°C. Here, a Purkinje potential preceded the local ventricular electrogram by 30 ms. Application of RF led to prompt interruption of the tachycardia, which was subsequently non-inducible following programmed ventricular stimulation. After a follow-up of 9 months, the patient remains free of palpitations and the Holter EKG revealed no VT episodes.

## Discussion

LVPFT is the most common form of idiopathic ventricular tachycardia originating from the left ventricle. It is characterized by a reentry mechanism using the slowly conducting verapamil-sensitive fibers and the posterior fascicle. The slowly conducting verapamil-sensitive fibers have decremental conduction properties, similar to the atrioventricular node [5]. The most common type of reentry uses verapamil-sensitive fibers as the antegrade limb and the posterior fascicle as the retrograde limb. Retrograde activation in the posterior fascicle occurs from the apical septum to basal septum [6]. The activation mapping performed during VT in the present case supports existing data from the literature, demonstrating that the reentry circuit does not need anterograde conduction through the posterior fascicle to support reentry, as the patient had left posterior fascicular block during sinus rhythm. Retrograde conduction can still be present during antegrade block; therefore, the fascicle can be used as the retrograde limb of the reentry circuit.

Kuo et al. [7] also showed that RF ablation of LVPFT did not result in fascicular block, concluding that the reentry circuit involves the tissue adjacent to posterior fascicle and not the fascicle itself. Morishima et al. [8] suggested that the posterior fascicle could be just a bystander to the reentry circuit. They used information from a sinus beat that selectively captured the posterior fascicle and performed entrainment mapping of the circuit. The recent report of Maeda et al. [9] elegantly demonstrated that the posterior fascicle was indeed a bystander, representing neither the antegrade nor retrograde limb of the circuit.

The anatomic basis of this arrhythmia has attracted considerable interest over time. It was discovered that some patients with LVPFT have a false tendon or fibromuscular band in the left ventricle. The exact mechanism by which this fibromuscular band contributes to the tachycardia is incompletely understood; some authors suggest electrical conduction through the fibromuscular band, others believe that stretch produced by this false tendon in the Purkinje network could provide the necessary conditions to tachycardia initiation [9, 10].

In most cases, radiofrequency ablation can be performed successfully [11]. However, when the tachycardia is non-inducible during the electrophysiological study, the site of ablation cannot be identified. In these cases, electroanatomical mapping can provide a more precise site for RF ablation: the mid-septum or the apical septum of the left ventricle [12].

In the study of Chen et al., mapping and linear ablation were performed during sinus rhythm using a three-dimensional mapping system. They demonstrated that by creating a linear lesion perpendicular to the posterior left fascicle, the tachycardia substrate is modified enough so that it can no longer be induced, with the cost of a left posterior fascicular block [13].

## Conclusion

When suspicion of LPFVT on the 12-lead ECG exists, the diagnosis and the mechanism can be clarified using the electrophysiological study. First-line pharmacological treatment is Verapamil, due to the involvement of a verapamil-sensitive zone in the reentry circuit. Catheter ablation is an effective treatment method and is recommended when symptoms are severe or when pharmacological treatment is ineffective/poorly tolerated, or antiarrhythmic drugs are not desired by the patient. The present case is particular by the presence of left posterior block during sinus rhythm, which demonstrates that the reentry circuit does not need the posterior fascicle as an antegrade limb for the perpetuation of the arrhythmia.

## Consent

The patient, who is a medical student, gave his consent for publication of medical information and images concerning his case. His real name is not provided.

## Abbreviations

VT: ventricular tachycardia
LPFVT: left posterior fascicular ventricular tachycardia
i.v.: intravenously
